# Coronavirus Disease Pandemic (COVID-19): Challenges and a Global Perspective

**DOI:** 10.3390/pathogens9070519

**Published:** 2020-06-28

**Authors:** Yashpal Singh Malik, Naveen Kumar, Shubhankar Sircar, Rahul Kaushik, Sudipta Bhat, Kuldeep Dhama, Parakriti Gupta, Kapil Goyal, Mini P. Singh, Ujjala Ghoshal, Mohamed E. El Zowalaty, VinodhKumar O. R., Mohd Iqbal Yatoo, Ruchi Tiwari, Mamta Pathak, Shailesh Kumar Patel, Ranjit Sah, Alfonso J. Rodriguez-Morales, Balasubramanian Ganesh, Prashant Kumar, Raj Kumar Singh

**Affiliations:** 1 Division of Biological Standardization, ICAR-Indian Veterinary Research Institute, Izatnagar, Bareilly, Uttar Pradesh 243122, India; shubhankar.sircar@gmail.com (S.S.); sudiptabhat1991@gmail.com (S.B.); 2 ICAR-National Institute of High Security Animal Diseases, OIE Reference Laboratory for Avian Influenza, Bhopal, Madhya Pradesh 462 022, India; navyog.yadav84@gmail.com; 3 Laboratory for Structural Bioinformatics, Biosystems Dynamics Research Center, Riken 250-0047, Japan; rahul.kaushik@riken.jp; 4 Division of Pathology, ICAR-Indian Veterinary Research Institute, Izatnagar, Bareilly, Uttar Pradesh 243122, India; mamtapathak48@gmail.com (M.P.); shaileshpatel624@gmail.com (S.K.P.); 5 Medical Microbiology, Department of Virology, PGIMER, Chandigarh 160012, India; parakritii@gmail.com (P.G.); kapilgoyalpgi@gmail.com (K.G.); minipsingh@gmail.com (M.P.S.); 6 Department of Microbiology, Sanjay Gandhi Postgraduate Institute of Medical Sciences, Raebareli Road, Lucknow, Uttar Pradesh 226014, India; ujjalaghoshal8@gmail.com; 7 Zoonosis Science Center, Department of Medical Biochemistry and Microbiology, Uppsala University, SE-75 123 Uppsala, Sweden; elzow005@gmail.com; 8 Department of Clinical Sciences, College of Medicine, University of Sharjah, Sharjah 27272, UAE; 9 Division of Epidemiology, ICAR-Indian Veterinary Research Institute, Izatnagar, Bareilly, Uttar Pradesh 243122, India; 10 Sher-E-Kashmir University of Agricultural Sciences and Technology of Kashmir, Shalimar, Srinagar, Jammu and Kashmir 190025, India; 11 Department of Veterinary Microbiology and Immunology, College of Veterinary Sciences, UP Pandit Deen Dayal Upadhayay Pashu Chikitsa Vigyan Vishwavidyalay Evum Go-Anusandhan Sansthan (DUVASU), Mathura, Uttar Pradesh 281001, India; ruchi.vet@gmail.com; 12 Department of Microbiology, Tribhuvan University Teaching Hospital, Institute of Medicine, Kathmandu P.O. BOX 1524, Nepal; ranjitsah57@gmail.com; 13 Public Health and Infection Research Group, Faculty of Health Sciences, Universidad Tecnologica de Pereira, Pereira 660001, Colombia; arodriguezm@utp.edu.co; 14 Grupo de Investigacion Biomedicina, Faculty of Medicine, Fundacion Universitaria Autonoma de las Americas, Pereira, Risaralda 660003, Colombia; 15 Laboratory Division, Indian Council of Medical Research -National Institute of Epidemiology, Ministry of Health & Family Welfare, Ayapakkam, Chennai, Tamil Nadu 600077, India; ganeshvirologist@yahoo.co.in; 16 Amity Institute of Virology and Immunology, J-3 Block, Amity University, Sector-125, Noida, Uttar Pradesh 201303, India; pkumar18@amity.edu; 17 Division of Veterinary Biotechnology, ICAR-Indian Veterinary Research Institute, Izatnagar, Bareilly, Uttar Pradesh 243122, India; rks_virology@rediffmail.com

**Keywords:** coronavirus, pandemic, SARS-CoV-2, COVID-19, pathobiology, diagnosis, vaccines, therapeutics

## Abstract

The technology-driven world of the 21st century is currently confronted with a major threat to humankind, represented by the coronavirus disease (COVID-19) pandemic, caused by the severe acute respiratory syndrome, coronavirus-2 (SARS-CoV-2). As of now, COVID-19 has affected more than 6 million confirmed cases and took 0.39 million human lives. SARS-CoV-2 spreads much faster than its two ancestors, SARS-CoV and Middle East respiratory syndrome-CoV (MERS-CoV), but has low fatality rates. Our analyses speculate that the efficient replication and transmission of SARS-CoV-2 might be due to the high-density basic amino acid residues, preferably positioned in close proximity at both the furin-like cleavage sites (S1/S2 and S2′) within the spike protein. Given the high genomic similarities of SARS-CoV-2 to bat SARS-like CoVs, it is likely that bats serve as a reservoir host for its progenitor. Women and children are less susceptible to SARS-CoV-2 infection, while the elderly and people with comorbidities are more prone to serious clinical outcomes, which may be associated with acute respiratory distress syndrome (ARDS) and cytokine storm. The cohesive approach amongst researchers across the globe has delivered high-end viral diagnostics. However, home-based point-of-care diagnostics are still under development, which may prove transformative in current COVID-19 pandemic containment. Similarly, vaccines and therapeutics against COVID-19 are currently in the pipeline for clinical trials. In this review, we discuss the noteworthy advancements, focusing on the etiological viral agent, comparative genomic analysis, population susceptibility, disease epidemiology and diagnosis, animal reservoirs, laboratory animal models, disease transmission, therapeutics, vaccine challenges, and disease mitigation measures.

## 1. Introduction

**Figure 1:**
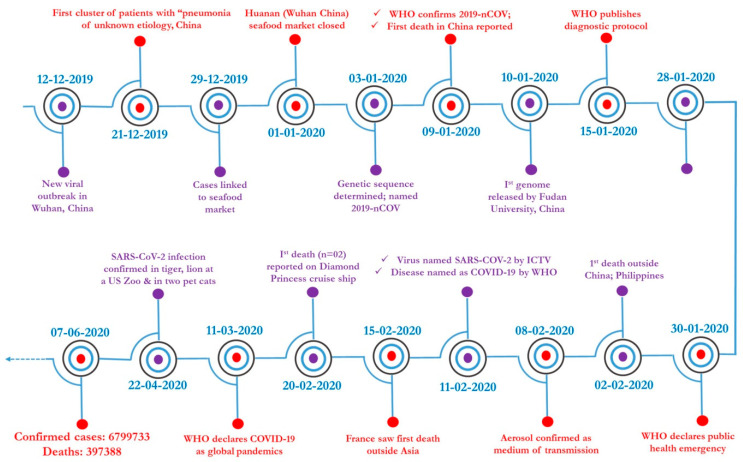
The timeline of significant events of COVID-19 caused by SARS-CoV-2. Major events, starting from the first report of novel CoV from Wuhan, China—including the declaration of COVID-19 as a worldwide pandemic by the WHO, and the situation as of June 7, 2020—have been depicted in the timeline format.

The ongoing global threat, declared a pandemic by international health agencies, originated from a newly emerging coronavirus (CoV), designated as severe acute respiratory syndrome coronavirus-2 (SARS-CoV-2) [1]. A live animal market in the Hubei Province of Wuhan in Mainland China was identified as the first epicenter of the pandemic [2]. Initially, an outbreak was suspected when a cluster of patients was admitted to local health care facilities with complaints of fever, cough, dyspnea, and fatigue, resembling symptoms common to viral pneumonia [3]. The first case of respiratory illness of unknown etiology occurred on 8 December 2019, as shown in Figure 1. Consequently, a cluster of patients reported respiratory illness to the local health care facilities, and on 30 December 2019, the Chinese National Health Commission (CNHC) suspected an outbreak of pneumonia of unknown etiology in Wuhan. With the increasing number of cases in neighboring countries and other areas worldwide, the disease spread to several countries worldwide and it was declared a Public Health Emergency of International Concern by the World Health Organization (WHO) on 31 January 2020, as shown in Figure 1. The epidemic intensified and spread without boundaries and affected more than 215 countries and a few international conveyances, which led the WHO to declare COVID-19 as a pandemic on 11 March 2020 [4].

As of 21 May 2020, 4,893,186 cases have been reported, with 323,256 deaths, of which 1,501,876 confirmed cases and 90,203 deaths were from the United States of America, followed by Brazil (271,628; 17,971), the United Kingdom (248,297; 35,704), Spain (232,555; 27,888), Italy (227,364; 32,330), France (141,312; 28,081), and China (84,507; 4645) [5]. Notably, all age groups show susceptibility to SARS-CoV-2 infection, from newborns to the elderly [6]. The average age noted for SARS-CoV-2 infection susceptibility is 55.5 years, while that for the case fatality rates (CFRs) is 75 years [7,8]. However, the CFR varies among different age groups and is highest (14.8%) among the elderly (>80 years of age). The incidences and CFR are reportedly higher in males (67.67% and 2.8%, respectively) than in females, owing to the higher frequency of engagement in outdoor activities and smoking status in males [9]. Immunocompromised individuals and patients with comorbidities are at a higher risk. The most common reported comorbidities in COVID-19 cases include cardiovascular diseases (10.5%), diabetes (7.3%), chronic respiratory infections (6.3%), hypertension (6%), and malignancies (5.6%) [8]. As per the WHO observations, the COVID-19 epidemic peaked and plateaued between 23 January and 2 February 2020, in China, and then followed a declining trend, paving a path for the elimination of the epidemic. However, the WHO has simultaneously stated that other countries are reporting a continuous increase in the number of cases, with the USA reporting the highest increase in daily deaths.

The definitive origin of SARS-CoV-2 remains undetermined. However, initial estimations implicate Wuhan as the epicenter and the live animal seafood market as the source of this outbreak, owing to the involvement of different animals, including wild and domestic animals, in close proximity to each other [10]. The postulation was primarily based on the significant genomic similarity between SARS-CoV-2 and bat or pangolin CoVs. The first patient to be reported in Wuhan did not visit the seafood market, and molecular analyses of SARS-CoV-2 genomic sequences suggest its origin is traced back to late November [11,12]. These findings raised specific questions pertinent to the COVID-19 epidemic and the Wuhan seafood market. The well-established human transmission of the outbreak emphasizes the likelihood of the better adaptability of the virus in transmission dynamics [3,11,13].

The events leading to the emergence of a virus capable of infecting a new host have occurred previously in CoV infections [14]. Earlier, reports suggested the emergence of SARS-CoV by a recombination between bat SARS-like CoVs, followed by mutations in civet CoVs prior to its spillover. Similarly, the Middle East respiratory syndrome (MERS)-CoV also circulated and acquired mutations in camels for approximately 30 years before the incidence of MERS in the Middle East [15,16], indicating the adaptation of CoVs to different hosts before their spillover to humans [17]. SARS-CoV-2 is suggested to be a chimera of two different viruses, a bat-CoV (RaTG13) and a pangolin-CoV [18].

Researchers have put forth their best efforts in developing effective treatments and vaccine candidates, and have succeeded to a significant extent in identifying several repurposed drugs, monoclonal therapeutic antibodies, and vaccine candidates. However, many such treatment options are in the different stages of pre-clinical and clinical development. To date, no approved, specific antiviral agent or vaccine against SARS-CoV-2 is available for human use. The development of next-generation effective diagnostics has undergone a great leap. This review highlights the significant progress made in the measures for the resolution of the COVID-19 pandemic, using virus genomic analysis, the assessment of global epidemiological trends, transmission patterns, the present-day status of therapeutics, and public health preparedness plans for controlling the wider dissemination of the disease.

## 2. Previous Human CoV Epidemics

Usually, infections caused by the *Betacoronaviruses* are mild to asymptomatic [19,20]. Since the first report of CoV in the 1960s, humans have been affected by CoV infection. To date, six strains of CoVs, causing mild to severe respiratory illnesses in humans, have been identified [21]. Of these, four CoVs (HCoV-NL63, HCoV-229E, HCoV-OC43, and HCoV-HKU1) cause mild symptoms in humans. Contrarily, two highly pathogenic CoVs caused outbreaks of severe respiratory illness due to zoonotic transmission in humans, namely the severe acute respiratory syndrome (SARS) and MERS.

### 2.1. SARS-CoV

The first incidence of SARS-CoV infection was reported on 16 November 2002, in the coastal Guangdong province of southeast China bordering Hong Kong and Macau. This infection spread across 31 countries at a moderate speed. The virus was predicted to originate from bats and civet cats with animal-to-human (zoonotic) and human-to-human modes of transmission, as shown in Table 1. A winter season predilection was also noted in the pattern of this epidemic. After adopting strict public health measures, consisting of quarantine and the lockdown of air traffic, the disease was finally contained by 5 July 2003 [22]. During this period, SARS-CoV infected 8422 humans and caused 916 deaths at a CFR of 10.87%. Since then, SARS has phased out, and the outbreak was managed.

### 2.2. MERS-CoV

Since the first occurrence of MERS in Saudi Arabia in June 2012, sporadic cases are still reported, primarily in the Arabian Peninsular region, and MERS has spread to more than 27 countries worldwide. Notably, the CFR in MERS is the highest (34.77%) among all CoVs. To date, it has infected 2496 humans with the deaths of 868 patients. The disease was reported in Saudi Arabia, the UAE and the Republic of Korea, as shown in Table 1. A few travel-related cases have been reported in Europe in individuals with a travel history to the Arabian Peninsular countries. The source of viral origin was traced to bats, and camels were the intermediate hosts for the virus, which followed animal-to-human (zoonotic disease) and human-to-human modes of virus transmission [19,23]. 

### 2.3. SARS-CoV-2

The first case of SARS-CoV-2 infection was reported in the first week of December 2019 in Wuhan City, Hubei Province, China. Using deep sequencing methods, the causative agent for this deadly pandemic was identified as a new member of the *Betacoronavirus* genus [24,25]. The newly identified CoV had an 82% sequence identity with the human SARS-CoV [26]. It was initially designated as 2019-novel coronavirus (2019-nCoV). Later, the International Committee on Taxonomy of Viruses officially named it as the severe acute respiratory syndrome coronavirus-2 (SARS-CoV-2), owing to its significant genetic relatedness to SARS-CoV. Subsequently, the WHO named the disease caused by SARS-CoV-2 as coronavirus disease-2019 (COVID-19) on 11 February 2020, as shown in Figure 1.

## 3. SARS-CoV-2 Taxonomy, Structure, and Genomics

SARS-CoV-2 belongs to the family *Coronaviridae*, subfamily *Orthocoronavirinae*, under the order *Nidovirales*. The *Orthocoronavirinae* subfamily consists of four genera, namely *Alphacoronavirus*, *Betacoronavirus*, *Gammacoronavirus*, and *Deltacoronavirus* [27]. SARS-CoV-2 is a *Betacoronavirus*. *Betacoronaviruses* are further subdivided into five subgenera, namely *Embecovirus*, *Hibecovirus*, *Merbecovirus*, *Nobecovirus*, and *Sarbecovirus*. Phylogenetically, it forms a discrete lineage within the subgenus of the *Sarbecovirus* [28]. CoVs, as their name suggests, have a crown-shaped appearance, as observed under the electron microscope, owing to the spikes on the surface of the virion.

SARS-CoV-2 is a 0.12 µm, enveloped, non-segmented virus, and its genome comprises a single-stranded positive-sense RNA (+ssRNA) of ≈29.8 kb [29,30]. The genome is arranged in a structure typical to coronaviruses in the following order: 5′-ORF1a/ab-Spike (S)-Envelope (E)-Membrane (M)-Nucleocapsid (N)−3′; however, it lacks the hemagglutinin–esterase gene, similar to bat SARS-like-CoVs, human SARS-CoVs, and MERS-CoVs. Fourteen open reading frames (ORFs) have been identified that encode twenty-seven proteins, both structural and non-structural proteins (nsps). At the 5′ terminus, the first ORF1a/ab, which occupies approximately two-thirds of the entire genome length, encodes the pp1a and pp1ab proteins, respectively, and both collectively comprise 15 non-structural proteins (nsp 1 to 10 and nsp 12 to 16). The functions of the nsps of a typical coronavirus and SARS-CoV-2 are listed in Supplementary Table S1 [26,31]. The ORFs toward the 3′- terminus encode at least four major structural proteins: the spike (S), matrix membrane (M), envelope (E), and nucleocapsid (N) proteins. Besides, accessory proteins (3a, 6, 7a, 7b, 8, and 10) are also encoded by the genome of SARS-CoV-2 Wuhan HU1. The 5′- and 3′- UTR sequences of SARS-CoV-2 have ≈83.0% nucleotide identity with other members of the *Betacoronavirus* genus. The genomes of SARS-CoV, SARS-CoV-2, and bat-SARS CoV are considerably similar. The RNA secondary structures in the 5′-UTR were more identical to those of SARS-CoV than to the bat SARS-like CoV; however, the 3′-UTR was more conserved compared to other related *Betacoronaviruses* [26].

**Figure 2:**
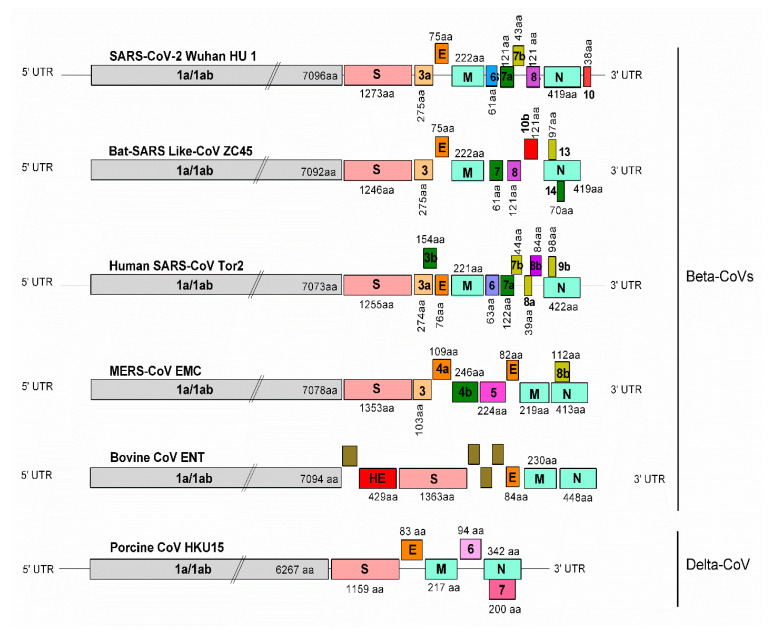
Genome organization of SARS-CoV-2 and its comparison with other coronaviruses. The coding regions of beta-CoVs, SARS-CoV-2 Wuhan-HU-1 (NC_045512.2; 29,903 bps), bat SARS-like-CoVZC45 (MG772933.1; 29,802 bps), human SARS-CoV Tor2 (AY274119.3; 29,751 bps), MERS-CoV EMC (NC_019843.3; 30,119 bps), bovine CoV ENT (NC_003045.1; 31,028 bps), and a delta-CoV, the porcine CoV HKU15 (NC_039208.1; 25425 bps) are presented. The genomes consist of a 5′ untranslated region (5′ UTR), an open reading frame (ORF 1a/ab) encoding non-structural proteins (nsps), structural proteins, hemagglutinin–esterase, spike (S), membrane (M), envelope (E), and nucleocapsid (N) proteins, several accessory proteins, and a 3′ untranslated region (3′ UTR). The lengths of the ORFs, nsps, and accessory proteins are not drawn to scale.

A detailed comparative genomic organization of important and related *Betacoronavirus* members, including a *Deltacoronavirus* as well, is provided in Figure 2. At the genomic level, the sequence homology of SARS-CoV-2 with MERS-CoV is lower (50–51.8%) than that with SARS-CoV (79%) [32]. Phylogenetically, SARS-CoV-2 is most similar to the bat SARS-like-CoVZC45 concerning most genes [33,34]. Even at the amino acid level, SARS-CoV-2 is relatively similar to bat SARS-like-CoVZC45, although there exist certain notable differences. For example, the 6, 7b, and 10 proteins are present in SARS-CoV-2, whereas these are absent in bat SARS-like CoVZC45. Compared to SARS-CoV, the sequence for 8a is absent in the SARS-CoV-2 genome; the sequence for 3b is shorter, while that for 8b has an extra 73 amino acids in SARS-CoV-2 [29]. The spike protein-encoding gene in SARS-CoV-2 (1273 amino acids) is longer than those of bat SARS-like-CoVZC45 (1246 amino acids) and SARS-CoV Tor2 (1255 amino acids). Certain accessory proteins, such as the 6 and 7b proteins of SARS-CoV Tor2, are similar to those in SARS-CoV-2, except for a marginal variation in protein length, as shown in Figure 2. The majority of the accessory proteins in MERS-CoV EMC are distinctive, including a longer spike protein (1353 amino acids), compared to those in SARS-CoV-2. In comparison to SARS-CoV-2, bovine CoV ENT has the longest spike protein (1363 amino acids) and a unique hemagglutinin–esterase gene, while porcine CoV HKU15 (a delta-CoV) has the shortest spike protein (1159 amino acids) and lacks several accessory proteins. Hence, it would be interesting to characterize the spike and accessory proteins of SARS-CoV-2 and study the role of these proteins in the pathogenesis and transmissibility of the virus.

**Figure 3:**
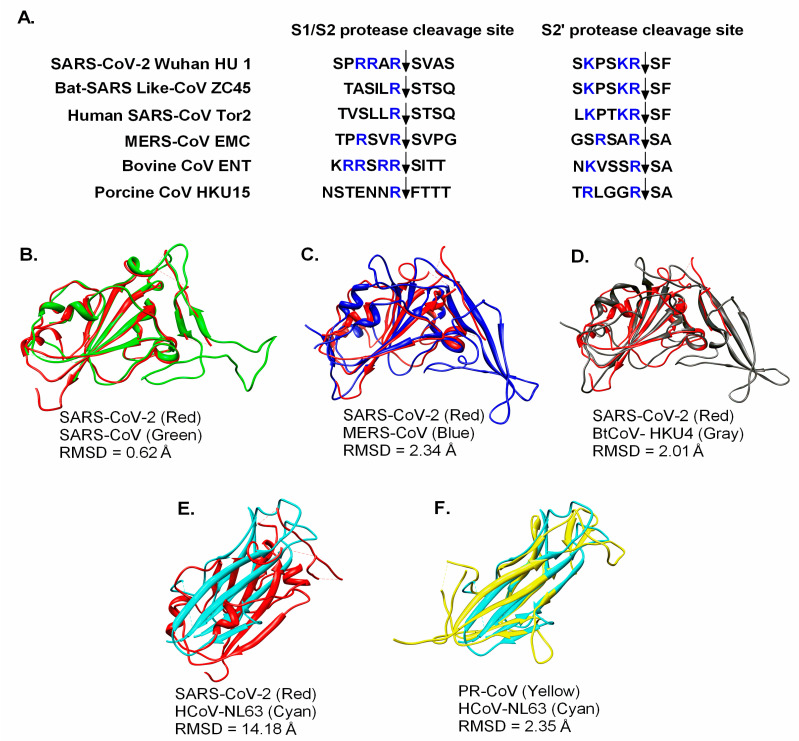
Comparative sequence and structural analyses of the SARS-CoV-2 spike protein. (**A**) The cleavage sites of SARS-CoV-2 spike proteins (S1/S2 and S’ protease cleavage sites) and their comparison with those in other coronaviruses. The superimposition of the receptor-binding domain (RBDs) of SARS-CoV-2 (pdb: 6VSB) on (**B**) human SARS-CoV (pdb: 2AJF), (**C**) MERS-CoV (pdb: 4KR0), (**D**) BtCoV-HKU4 (pdb: 4QZV), and (**E**) HCoV-NL63 (pdb: 3KBH). (**F**) The superimposition of the RBDs of porcine respiratory CoV (pdb: 4F5C) on HCoV-NL63 (pdb: 3KBH). RMSD: Root Mean Square Deviation. UCSF Chimera was used for the superposition and visualization of the RBDs of the different CoVs [40].

The trimeric spike (S) glycoprotein, the most important determinant of host cell tropism and transmission capability, is functionally composed of two subunits, the S1 (receptor binding) and S2 (cell membrane fusion) [33,35]. The S2 subunit of SARS-CoV-2 is comparatively conserved and closely related to that of bat SARS-like-CoVZC45, while the S1 subunit had only 70% identity with that of bat SARS-like-CoVZC45 [26,34]. During infection, the S protein is proteolytically processed by host cell proteases at the S1/S2 cleavage site and, following cleavage, the S protein is cleaved into the N-terminal S1 subunit and C-terminal S2 subunit. The S1 subunit comprises a signal peptide, an N-terminal domain, and a receptor-binding domain (RBD). In contrast, the S2 subunit comprises a conserved fusion peptide, a second proteolytic site (S2′), followed by an internal fusion peptide, heptad repeats 1 and 2, a transmembrane domain, and a cytoplasmic domain [36]. The cleaved S protein remains in the metastable pre-fusion conformation [37,38]. After the virus enters the host cell, a second cleavage (at the S2′ cleavage site) occurs for membrane fusion, mediated by the endolysosomal proteases [33]. Since furin is highly expressed in the lungs, the furin-like cleavage sites (S1/S2 and S2′) appear to be critical for the activation of the S protein and, therefore, for the efficient entry of virus [36,39]. On comparing these sites, we observed a typical furin-like cleavage site rich in basic amino acid residues (SP**RR**A**R**SVAS) in SARS-CoV-2 that is absent in bat SARS-like-CoVZC45 (TASIL**R**STSQ) and human SARS-CoV Tor2 (TVSLL**R**STSQ), as shown in Figure 3. However, the S2′ cleavage site patterns (X**K**PX**KR**SF) in all three viruses were considerably similar.

Similarly, furin might cleave the MERS-CoV EMC spike protein less efficiently at the S2′ cleavage site, owing to the lack of typical basic amino acid residues observed in SARS-CoV-2, bat SARS-like-CoVZC45, and human SARS-CoV Tor2. Although the S1/S2 cleavage site in the bovine CoV ENT (K**RR**S**RR**SITT) spike protein is rich in basic amino acids, its S2′ cleavage site (N**K**VSS**R**SA) lacks the typical basic amino acid residues, as shown in Figure 3. The porcine CoV HKU15 contains a monobasic cleavage site (NSTENN**R**FTTT), which could lead to low-efficiency cleavage. Based on the findings from these comparative studies, we speculate that the high-density basic amino acid residues, preferably positioned in close proximity at both cleavage sites (S1/S2 and S2′), might have facilitated the efficient replication and transmission of SARS-CoV-2. However, these propositions need to be validated by further experimental investigations.

## 4. CoV Receptors

Host cell receptors and viral surface glycoprotein/ligand binding serve as the most important determinants of host range, cross-species transmission, and antiviral targeting inventions [41,42]. As shown in Table 2, CoVs can infect a wide range of host species, including humans, animals, and birds. CoVs from four genera recognize at least five different receptors and, therefore, exhibit an intricate host receptor recognition pattern. The differential usage of host receptors by different CoVs indicates the structural diversity in the RBDs of the S glycoprotein. However, CoV infections in humans are primarily driven by interactions between the envelope-anchored spike glycoprotein (S protein) of CoV and the host cell receptor, the angiotensin-converting enzyme 2 (ACE2) [43,44,45]. Viruses from the same genus can recognize different receptors (for example, SARS-ACE2 and MERS-DPP4), while CoVs from different genera can bind to the same receptor as well, such as ACE-2 (HCoV-NL63 and SARS). The key features of the external subdomain of RBDs need to be investigated further to elucidate their role in receptor usage, transmission, and the pathogenesis of SARS-CoV-2.

## 5. SARS-CoV-2 Persistence/Susceptibility on Different Surfaces

With the exponential increment in SARS-CoV-2 cases globally, the non-availability of effective and specific antiviral treatment, and the lack of specific vaccines, the primary focus has been on minimizing contact with infected persons, asymptomatic carriers, or contaminated surfaces to contain the spread of the virus. Given this, we reviewed the available literature on viral persistence on different contaminated surfaces that we commonly come into contact with daily. SARS-CoV-2 has been shown to persist in aerosols for up to three hours, with a decline in the infectious virus titer from 10^3.5^ to 10^2.7^ TCID_50_ per liter of air [46]. Meanwhile, SARS-CoV-2 was found to be retained more stably on plastic and stainless steel and could retain infectivity for up to 72 h when present on these contaminated surfaces. However, the infective titer reduced significantly from 10^3.7^ to 10^0.6^ TCID_50_ on plastic surfaces and from 10^3.7^ to 10^0.6^ TCID_50_ on stainless steel surfaces. On copper surfaces, both SARS-CoV-2 and SARS-CoV-1 were rendered non-viable after four and eight hours, whereas on cardboard surfaces, no viable viruses were detected after 24 and 8 h, respectively [46]. Other CoVs, such as MERS-CoV, remained viable for up to 48 h on plastic and stainless steel surfaces [47]. A limited number of low pathogenicity human CoVs, such as the HCoV-229E and HCoV-OC43, were found to be viable from 2–9 h up to six days on different surfaces, such as aluminum, glass, plastic, PVC, silicon rubber, latex gloves, ceramic, and Teflon [48,49,50].

Owing to the significant number of COVID-19 cases worldwide, and the lack of effective remedies for this disease, the WHO is constantly endorsing and promoting better personal hygiene practices. Social and physical distancing, as well as self-isolation, are recommended to contain the spread of the virus. Therefore, the WHO recommended two hand rub formulations based on ethanol and 2-propanol [51]. Such alcohol-based hand rub formulations were found to be effective in inactivating SARS-CoV-2 [52].

## 6. Phylogenomic Analysis of SARS-CoV-2 and Related CoVs

We analyzed the spike gene sequences (*n* = 38) of SARS-CoV-2, which were retrieved from the NCBI database. The spike gene sequences of CoVs, representing different subgenera of *Betacoronavirus*, including SARS-CoV and MERS-CoV, were also retrieved. Based on the BLASTn analysis, certain strains, similar to bat SARS-like-CoVs and bat-CoVs, were also included in this analysis. The nucleotide and amino acid percentage similarities between these CoVs were calculated using the MegAlign software from DNASTAR. The phylogenetic analyses of spike genes from different CoVs were conducted using the GTR + G substitution model and the Maximum Composite Likelihood method. The tree was visualized using iTOL, an online tree visualization tool [53].

The whole-genome sequence of SARS-CoV-2 derived from isolates from different countries showed a high similarity of 99 to 100% at both nucleotide and amino acid levels, suggesting the stability of the viral genome. To determine the similarity index between SARS-CoV-2 and other members of *Betacoronavirus,* we analyzed the spike protein sequences of all subgenera. The reference SARS-CoV-2 strain (Wuhan-Hu-1/NC_045512) was closely associated with a bat-CoV (RaTG13) from Yunnan, China, with 93.1 and 97.67% nucleotide and amino acid sequence similarity, respectively, as shown in Table 3. A pangolin-CoV strain MP789 from China also had a similarity index of 89.7 and 89.78% in nucleotide and amino acid sequences with the reference strain, respectively. Besides, two bat SARS-like-CoVs (bat-SL-CoVZC45/MG772933 and bat-SL-CoVZXC21/MG772934) were also closely related to the novel SARS-CoV-2, as shown in Table 3.

The clustering of SARS-CoV-2 with the bat and pangolin CoVs provided evidence of its possible origin from these animals. Another member of the subgenus *Sarbecovirus*, SARS-CoV, was distantly related to SARS-CoV-2, as shown in Table 3.

**Figure 4:**
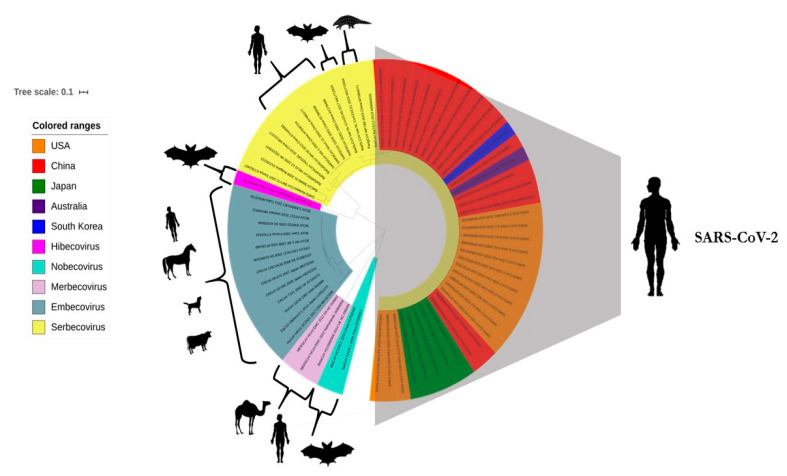
Maximum likelihood tree of *Betacoronaviruses*, including SARS-CoV-2. The phyloanalysis included SARS-CoV-2 originating from China, the USA, Australia, Japan, and South Korea. Different subgenera of *Betacoronaviruses* have been labeled with different colors according to the color legend on the left side of the tree. Major species of each subgenus have been depicted in front of each clade.

Based on the spike gene phylogenetic analysis, it was observed that SARS-CoV-2 originating from different countries formed a separate clade with other members of *Sarbecovirus*. It was found that the bat-CoV (RaTG13/MN996532), pangolin-CoV (MP789/MT084071), and two bat SARS-like-CoVs (bat-SL-CoVZC45/MG772933 and bat-SL-CoVZXC21/MG772934) closely clustered with SARS-CoV-2, while other bat-CoVs and SARS-CoVs clustered within the *Sarbecovirus* subgenus clade, as shown in Figure 4. Similarly, CoVs from different subgenera, which included bat-CoVs belonging to *Hibecovirus* and *Nobecovirus,* human and camel MERS-CoV belonging to *Merbecovirus,* and human, canine, bovine, and equine CoVs belonging *Embecovirus* subgenera, clustered separately, as shown in Figure 4.

## 7. Epidemiology of SARS-CoV-2 Infection

The Wuhan seafood market in China was implicated as the epicenter of the COVID-19 pandemic, and China was marked as the index country. Seven cases of pneumonia of unknown etiology were reported in Wuhan from 8–18 December 2019. The first cluster of such patients was identified on 21 December 2019. Pneumonia of unknown etiology is defined as an illness without an identified causative pathogen that fulfills the following criteria: fever (≥38 °C), radiographic evidence of pneumonia, low or average white-cell count or low lymphocyte count, and no symptomatic improvement after antimicrobial treatment for 3 to 5 days following standard clinical guidelines [11,54]. The patients presented with a severe acute respiratory infection and some developed acute respiratory distress syndrome (ARDS) and associated complications [55].

### 7.1. Transmission

Information about the mode of transmission of SARS-CoV-2 is continuously evolving. Transmission primarily occurs via the person-to-person route through respiratory droplets (>5–10 μm in diameter) and fomites. Droplet infection is associated mainly with coughing, sneezing, and talking to an infected person, and the virus is transmitted to humans through direct contact with mucus membranes. Transmission can also occur by touching an infected surface, followed by contact with eyes, nose, or mouth. The travel range of the droplets is less than six feet (approximately two meters), and these are not retained in the air for long. Satisfactory evidence of the airborne transmission of SARS-CoV-2 remains unavailable [46]. Although clinically infected patients readily transmit the virus [14], asymptomatic infected patients can also transmit the virus, and therefore, these carriers should be tested to effectively contain the spread [11,56,57].

There is evidence of intestinal infection from SARS-CoV-2, and subsequent excretion through feces [56,58]. In China, out of ten pediatric COVID-19 patients without respiratory symptoms, eight were positive for fecal SARS-CoV-2 shedding; however, the nasopharyngeal samples from these subjects tested negative for the virus [59]. These findings were further supported by a study in which the virus was successfully cultured from feces samples [59,60]. However, there have been no reports of fecal–oral transmission of SARS-CoV-2 to date. Some reports suggest SARS-CoV-2 transmission through semen and the infection of SARS-CoV-2 in newborns; however, there is no evidence for vertical transmission or intrauterine infection in a COVID-19 patient during the late pregnancy stage [55].

### 7.2. Incubation Period

The incubation period for SARS-CoV-2 infection ranged from 2 to 14 days in human-to-human transmission, while, in some cases, it extended up to 24 days [57]. However, a median incubation period of 5-6 days has recently been reported by the WHO [61].

### 7.3. Basic Reproduction Number (R_0_)

The basic reproduction number plays a pivotal role in infectious disease epidemiology and indicates the risk a pathogen carries with respect to its spread. In other words, R_0_ provides information about the transmissibility of a virus and represents the number of new infections originating from an infected individual in a population. A recent comparison of different studies on the estimation of R_0_ for COVID-19 revealed that the estimated mean R_0_ for COVID-19 is 3.28, with a median of 2.79, which is higher than the WHO estimate of 1.95 [62]. However, considerable variability was noted, owing to the use of different methods, insufficient data, and the short onset time of the disease.

### 7.4. Population Susceptibility

There is no age limit for vulnerability to SARS-CoV-2 infection. However, cases with higher morbidity and chances of mortality have been reported in older adults. Individuals above 65 years of age or those who have comorbidities (such as hypertension, diabetes, cardiovascular diseases, and respiratory system diseases) are highly susceptible to the infection [25]. Women are less susceptible to SARS-CoV-2 infection than men and this is possibly due to different innate immunity, and factors related to sex chromosomes in women [63]. For example, the biallelic expression of TLR7 in women allows higher immune responses, while the immune regulatory genes encoded by X chromosomes cause comparatively less virus load and inflammation. Recently, studies have shown that SARS-CoV-2 infection in children (though less severely affected), may develop the Kawasaki disease-like forms [64]. However, it is still not clear whether Kawasaki disease-like forms are a result of SARS-CoV-2 infection alone or just SARS-CoV-2 induced aggravations in Kawasaki disease patients.

### 7.5. Global Scenario

**Figure 5:**
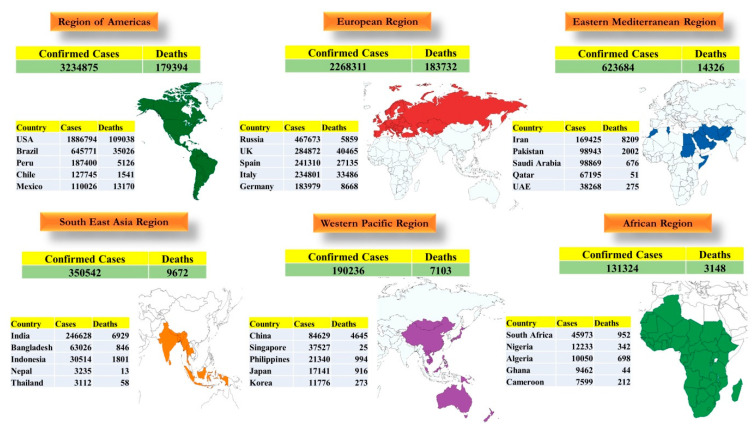
**Worldwide distribution map depicting COVID-19 cases and deaths in major world regions.** Six regions have been described by the WHO where the COVID-19 disease is present and are depicted on the map along with confirmed cases and deaths represented as of June 7, 2020.

COVID-19 has affected almost every country across the globe. On 31 December 2019, an “urgent notice on the treatment of pneumonia of unknown cause” was issued to the Wuhan Municipal Health Center. The market premises were disinfected, and the stallholders were instructed to wear masks. Till 3 January 2020, 44 case-patients with pneumonia of unknown etiology were reported to the WHO from China, although the cause was not identified. On 7 January 2020, the Chinese authorities identified a new type of CoV and shared its genetic sequence on 12 January 2020. On 9 January, the death of a 61-year-old man was the first reported case of death from COVID-19. The WHO published interim laboratory guidelines for the detection of SARS-CoV-2 [65]. As of 7 June, 2020, COVID-19 distribution was seen globally with large number of confirmed cases and deaths. The WHO defined six regions to distribute the COVID-19 disease (Figure 5).

Between 13–20 January 2020, Thailand (the first country after China to report a case), Japan, and the Republic of Korea, reported their respective first cases of COVID-19. On 20 January, the China CDC identified three different strains of this virus, confirming the mutation of the original virus. Six deaths were reported in Wuhan, of which four patients had underlying comorbidities [66].

Between 22 and 25 January, Macau, Hong Kong, Nepal, France, Australia, Malaysia, and Canada confirmed their first cases. As in China, the WHO assessed the risk to be significantly high at both regional and global levels. On 30 January 2020, the International Health Regulations Emergency Committee, WHO declared the outbreak as a public health emergency of international concern. Later on, the WHO named the virus as SARS-CoV-2, and the disease was named COVID-19 (coronavirus disease 2019). Thus far, SARS-CoV-2 has affected essentially all countries in the world, infected millions of people, and killed hundreds of thousands of people [67].

### 7.6. Socio-Economic Impact

The COVID-19 pandemic has resulted in the imposition of international travel restrictions in several countries, the cancellation of major sporting events, and the lockdown of borders, and has had a major impact on the global economy. It has also resulted in the closure of schools and universities worldwide (10 March, UNESCO). The stock markets and crude oil prices have declined severely, possibly the most drastic occurrence in recent years. Industries related to entertainment, ceremonial activities, hotels, and tourism are also affected. It is estimated that the global GDP loss is within the range of 1.0 to 2.7 trillion US dollars. The lockdown and other associated restrictions have severely affected the economic status and the well-being of families and communities. The major sectors affected by this outbreak are health, education, manufacturing, transportation, and trade, among others.

## 8. Clinical Profile

CoVs include viruses infecting humans and other animal species, such as birds, camels, cattle, bats, and cats. CoVs primarily affect the upper and lower respiratory tracts, and the enteric, hepatic, and neurological systems, with infections characterized by severe dyspnea and hypoxemia, renal dysfunction with lower urine output, tachycardia, altered mental status, and functional alterations of organs in animals and humans. Individuals infected with SARS-CoV-2 may be asymptomatic carriers or active patients with the clinical presentation of symptoms, such as fever, fatigue, dry cough, myalgia, dyspnea, severe respiratory distress syndrome, and acute cardiac injury [57,68,69]. There are three types of ARDS: mild, moderate, and severe ARDS. An increase in body temperature up to 39.0 °C (102.2 °F), accompanied by coarse breathing sounds from both lungs at auscultation are common clinical symptoms. Symptoms, such as headache, sputum production, hemoptysis, diarrhea, and pleuritic chest pain were also observed in rare cases. The infection more commonly affects people with cardiopulmonary or renal diseases, and pregnant females, infants, and the elderly (at a two-fold greater risk than the general population). There is no evidence to suggest greater susceptibility among children. Pregnant women are at greater risk for viral infections, as suggested by data from previous outbreaks; therefore, it is advisable to avoid contact with infected individuals [70].

## 9. Clinical Pathology and Immunopathobiology

### 9.1. Clinical Pathology

Based on the time of occurrence and intensity of symptoms, the infection has been categorized into three stages; mild, severe, and critical stage [71,72]. In the mild stage, mild pneumonia/no pneumonia develops, although symptoms of upper respiratory tract viral infection, marked by mild fever, dry cough, sore throat, nasal congestion, malaise, headache, and muscle pain, prevail [73]. In severely affected patients, the rate of respiration is above 30/min due to dyspnea, respiratory symptoms, such as cough and shortness of breath, or tachypnea, and oxygen saturation in the blood below 93%, indicating hypoxia, and these developments are observed within a time period of 24–48 h. The critical stage is marked by symptoms, such as severe pneumonia, septic shock, respiratory failure, cardiac arrest, and/or multiple organ failure, which may eventually lead to the death of the patient [74,75].

Radiological images indicate that two to five lobes of the lungs may be affected. The most common and pronounced lesions are marked by patchy ground-glass opacities and patchy consolidation in the mid, external, and sub-pleural areas of the lung [76]. A chest radiograph, computer tomographic (CT) scan, and/or lung ultrasound indicate the presence of bilateral opacities [55,77,78]. Thoracic CT scan imaging depicted bilateral ground-glass opacity and bilateral multiple lobular and sub-segmental areas of consolidation [79,80,81]. The pathological image of a COVID-19 patient presented bilateral diffuse alveolar damage with cellular fibromyxoid exudates. The cytopathic effects were marked by the presence of interstitial mononuclear inflammatory infiltrates, with a majority of lymphocytes and multinucleated syncytial cells, along with atypical enlarged pneumocytes containing large nuclei and amphophilic granular cytoplasm, in the intra-alveolar spaces. In some infected patients, microvascular steatosis and mild lobular and portal activity were recorded in hepatic tissues, while interstitial mononuclear inflammatory infiltrates were found in the cardiac tissues [8].

### 9.2. Immunopathobiology

Existing literature documents that patients suffering from severe SARS-CoV-2 infection might suffer from the cytokine storm syndrome that could eventually lead to death [82,83]. In a viral infection, cell-mediated immunity is triggered to overcome the infection; however, owing to the overproduction of cytokines, interferons, and other factors, such events can have drastic effects in certain patients [84]. Evidently, respiratory failure due to the ARDS is the leading cause of mortality, and, apart from this, secondary hemophagocytic lymphohistiocytosis is also induced in a limited number of adult COVID-19 patients. Usually, it is one of the less-recognized syndromes characterized by hyperinflammatory, hyperferritinemia, fulminant and lethal hypercytokinemia, cytopenias, coagulopathy, and thrombocytopenia, along with multiple organ failure. The severely affected group of COVID-19 patients presented with a marked increase in the levels of plasma pro-inflammatory cytokines, interleukin (IL)-2, IL-7, macrophage inflammatory protein 1-α, granulocyte colony-stimulating factor, tumor necrosis factor-α, interferon-γ inducible protein 10, and monocyte chemoattractant protein 1. Mortality in COVID-19 could be attributed to such hyperinflammatory reactions [82,85].

Clinical laboratory findings suggested leucopenia (white blood cell count less than 2.91 × 10^9^/l) and lymphopenia (lymphocyte count less than 1.0 × 10^9^/l), high erythrocyte sedimentation rate, altered myocardial zymogram with elevated levels of lactate dehydrogenase and creatinine kinase, increased levels of alanine aminotransferase or aspartate aminotransferase as indicators of irregular liver function, a rise in hypersensitive troponin I (hs-cTnI) as a sign of virus-induced heart tissue damage, and a marked increase in the concentration of serum C-reactive protein and D-dimer, hyperbilirubinemia, and acidosis [3,24].

Studies on the pathogenesis of COVID-19 have not been undertaken thus far; however, understanding the mechanisms underlying the pathogenesis of MERS-CoV and SARS-CoV may help delineate key factors associated with COVID-19 pathogenesis. Once the virus enters the cells through a specific interaction between the S protein and ACE-2, the antigen-presenting cells are likely to process the viral proteins through the major histocompatibility complex (MHC or human leukocyte antigen in humans). Following the MHC antigen presentation of SARS-CoV-2, both humoral and cellular immunity are activated, mediated by the antibody-producing B cells and virus-specific T lymphocytes. The seroconversion in COVID-19 patients was observed to occur at a similar point or slightly earlier than in SARS-CoV infection [86,87]. A recently released antibody profile against SARS-CoV-2 has indicated that the seroconversion rates for antibodies, IgM, and IgG were 93.1% (161/173), 82.7% (143/173), and 64.7% (112/173), respectively [88]. Studies on cellular immunity against SARS-CoV-2 are limited. A recent study reported a significant reduction in the levels of CD4 and CD8 T cells in the peripheral blood of SARS-CoV-2-infected patients [89]. Studies on COVID-19-recovered patients can help significantly in elucidating the role of specific memory T cell-mediated immune responses against SARS-CoV-2.

Increasing evidence suggests that fatalities resulting from SARS-CoV-2 infection could be primarily attributed to the ARDS [90,91], which may be associated with comorbidities and followed by multiple organ failure, leading to death [92]. ARDS is an immunopathological feature common to SARS-CoV-1, MERS-CoV, and the more recent SARS-CoV-2 infections [89,93]. ARDS occurs primarily in response to the cytokine storm, the fatal uncontrolled systemic inflammatory response, resulting from the release of an excess quantity of pro-inflammatory cytokines (IL-6, IL-1β, IL-2, IL-8, IL-17, G-CSF, GM-CSF, IP10, MCP1, MIP1α, and TNF) from immune effector cells during SARS-CoV-2 infection [91,93,94,95,96,97,98]. In this context, the increased levels of IL-6 were considered a reliable indicator of poor prognosis in the severe stage of the disease [98]. The cytokine storm triggers a severe attack by the immune system on the lung and the body, inducing ARDS and multiple organ failure, and finally leading to death in severe cases of SARS-CoV-2 infections, similar to that in SARS-CoV and MERS-CoV infections [89,94]. A recent study also supported the proposition that SARS-CoV-2 acts on lymphocytes and leads to a cytokine storm and a series of immune dysregulation events in the body [94,95,97].

## 10. Animal Reservoirs

The identification of animal reservoirs or intermediate host(s) and the evolution of SARS-CoV-2 is critical to our understanding of the molecular mechanism underlying interspecies transfer, and hence, devising effective control measures to prevent its further spread.

The preliminary whole-genome analyses of SARS-CoV-2 denoted a high genetic identity (96.2%) with a bat (BetaCoV/bat/Yunnan/RaTG13/2013) virus, detected in *Rhinolophus affinis* in the Yunnan province of China [99], leading to speculation that bats might act as a possible reservoir host. Notably, the homology modeling studies of RBD of SARS-CoV-2 suggest that it has a structure similar to that of SARS-CoV RBD, with few differences in the key residues at the amino acid level [33]. Another study based on synonymous codon usage bias suggested snakes as an intermediate host; however, this virus has not been detected in any snake species yet [100]. Later on, pangolins were found to be a potential intermediate host. The recent detection and whole-genome sequence analysis of SARS-CoV-2 from Malayan pangolins indicated a sequence identity of 85.5 to 92.4% with pangolin SARS-CoV-2, which is lower compared to that of bat CoV RaTG13 (96.2%). However, the RBD of the S protein from pangolin SARS-CoV-2 exhibited 97.4% amino acid similarity to that of SARS-CoV-2, which is higher compared to that of RaTG13 (89.2%) [101]. Furthermore, the SARS-CoV-2 RBD shares five identical key residues with that of pangolin-CoV, while it shares only one residue with that of RaTG13 [101]. This finding suggests that pangolins may act as a potential intermediate host. However, studies suggesting bats as a reservoir host and pangolins as an intermediate host for SARS-CoV-2 need to be investigated further.

Susceptibility in different animals has recently been evaluated through an experiment on SARS-CoV-2 infection, where SARS-CoV-2 was found to replicate efficiently in cats and ferrets, while it replicated poorly in pigs, dogs, chickens, and ducks [102]. Viral transmission via respiratory droplets was also noted in cats in this study. A dog and a tiger were reported to test positive for SARS-CoV-2 [103]. The gene sequence analysis of the viral isolate from a dog indicates that the dog might have acquired the infection from infected individuals. These findings suggest low chances of SARS-CoV-2 transmission from pet animals to the human population; however, further investigation is warranted.

## 11. Animal Models in SARS-CoV-2 Research

There is an ongoing search for a suitable animal model to study SARS-CoV-2 infection [104]. After the SARS outbreak, numerous inbred mouse strains were evaluated as models for the SARS-CoV disease with varying levels of results [105,106]. The genetically engineered mouse model, known as the humanized ACE2, was developed in response to the SARS outbreak. This model could be infected by SARS-CoV-2 and develop mild pneumonia. The other promising animal models for SARS-CoV-2 vaccine research could include hamsters and monkeys [107]. Recently, ferrets have been identified as an infection and transmission animal model for studying SARS-CoV-2. This finding may facilitate the development of SARS-CoV-2 drugs and vaccines [108].

## 12. Diagnosis of COVID-19

### 12.1. Sample Collection

For patients under investigation, the collection of upper respiratory samples (nasopharyngeal and oropharyngeal swabs) and lower respiratory samples (sputum, if possible) is recommended irrespective of the presentation. Nasopharyngeal swab, bronchoalveolar lavage, endotracheal aspirates or wash, oropharyngeal swab, and saliva are the preferred samples. Stool and urine samples can also be tested to exclude other unidentified modes of transmission [3]. Respiratory samples should be collected in viral transport medium with antibiotics and antifungals and transported in the cold chain. Sample collection and packaging have to be conducted using proper personal protective equipment. The triple packaging of samples is necessary to prevent spillage and should be conducted in a negative pressure room with Class II Biosafety cabinets to contain transmission. However, depending upon the available resources, certain modifications can be incorporated in the Standard Operating Procedures to minimize the risk. Lately, saliva samples have been found to test positive for SARS-CoV-2 in 91.7% of the symptomatic cases, and a declining trend in viral load has also been reported. This finding is of immense importance as the collection of saliva is a non-invasive technique, requiring less stringent conditions, and this can also be used as a screening tool [109]. Zhang et al. used anal swabs in addition to respiratory samples and found a higher number of positive cases using anal swabs than respiratory swabs, especially in the later stages of infection [60].

### 12.2. Diagnostic Technologies

Sensitive and specific diagnostic methods are always preferred, especially in the cases of epidemics or pandemics, for the rapid and accurate diagnosis of viruses. Molecular (RT-PCR) assays are fast, highly sensitive, and specific, and are gradually replacing conventional methods. According to data maintained by the Foundation for Innovative New Diagnostics (FIND), one of the WHO’s partners for the assessment of diagnostics, at least 143 COVID-19 molecular diagnostic kits are in commercial use worldwide [110]. Among these, 22 kits have already been assigned for emergency use authorization (EUA) by the U.S. Food and Drug Administration (FDA). The details of the selected kits are provided in Table 4.

The successful limitation of virus spread in Singapore, Taiwan, and Hong Kong, despite their geographical proximity to China, indicates that widespread and intense testing, combined with strict quarantine measures for positive and suspected cases, are crucial for combatting SARS-CoV-2. Large-scale screening is arduous with the currently used molecular tests; therefore, deployment of rapid point-of-care tests (POCTs) can act as a breakthrough in the current scenario [111]. In this context, a recent breakthrough in COVID-19 diagnosis was marked by the Xpert^®^ Xpress SARS-CoV-2 test (an RT-qPCR test) developed in the USA and reported as a rapid diagnostic test that provides result in 45 min. Moreover, it is an automated POCT with EUA from the FDA and enables the detection of SARS-CoV-2 in the nasal wash, nasopharyngeal swab, and aspirated samples [112]. The Abbott ID NOW™ COVID-19 test, a rapid point-of-care (POC) PCR isothermal test, which can deliver results in five minutes, has recently received the EUA from the FDA. Another FDA-approved POC test, a rapid IgM–IgG-combined antibody test for COVID-19, developed by Biomedomics (Becton Dickinson) can detect antiviral antibodies with 88.7% sensitivity and 90.6% specificity within 10–15 min [113]. These POC tests, which can be used at the community level, are likely to improve the testing capacities, and hence, would be pivotal in disrupting the SARS-CoV-2 transmission cycle. Recently, a CRISPR-based Specific High Sensitivity Enzymatic Reporter Unlocking (SHERLOCK) technique has been used to diagnose COVID-19 in less than an hour without using multiple instruments [114]. A study employed the combination of RT-PCR, CRISPR, and metagenomic next-generation sequencing (mNGS) for the detection of SARS-CoV-2 and confirmed the effective clinical diagnosis of COVID-19 [115].

### 12.3. Artificial Intelligence in COVID-19 Diagnosis

Artificial intelligence (AI) tools are being tested and applied for preliminary screening of possible early infections of SARS-CoV-2 among individuals. Based on the AI-based learning framework, individuals are being categorized as high, moderate, or minimal risk individuals [116]. However, multiple experts differ in their opinion over the data collected by these AI tools, as it is often considered sensitive, owing to concerns of national security [117].

The chest CT-based diagnosis, usually in COVID-19, has certain limitations in terms of differentiation between COVID-19 and community-acquired pneumonia. Therefore, a deep learning model for COVID-19 was developed and employed to distinguish the common community-acquired pneumonia from COVID-19 [118]. With a lack of point-of-care diagnostics, AI-driven tools can prove useful in determining the risk involved and the transmission dynamics among different population groups. Presently, owing to the involvement of active learning processes in AI tools, the rate of confidence in decision-making processes has increased [28,119]. Therefore, there is an urgent need to standardize protocols to develop AI-based devices that can be used during such disasters.

## 13. Vaccines and Therapeutics

### 13.1. Vaccine Development

The most common preventive and effective approach to decelerate the COVID-19 pandemic is the use of vaccines in humans. Currently, there are significant research efforts being made globally to develop safe and effective vaccines against SARS-CoV-2 infection [120]. Approximately 200 vaccine candidates have already been developed worldwide and are at different stages of clinical evaluation [121]. Most of these vaccine candidates have been developed using at least seven different approaches, which include mRNA vaccine, viral vector vaccine, DNA vaccine, recombinant protein-based vaccine, peptide-based, virus-like particles, killed vaccine, and live-attenuated vaccine. Currently, the COVID-19 Treatment and Vaccine Tracker, maintained by the Milken Institute School of Public Health, USA, provides comprehensive publicly-available information on all drugs and vaccines against SARS-CoV-2 that are currently being studied.

Notably, two vaccine candidates have reached phase II clinical trials. The first one, the mRNA-1273, a novel lipid nanoparticle encapsulated mRNA vaccine, which encodes a prefusion-stabilized form of the SARS-CoV-2 spike protein, has been jointly developed by the National Institute of Allergy and Infectious Diseases (NIAID), USA and Moderna, Cambridge, Massachusetts, as shown in Table 5 [122]. The potential advantage of such a vaccine is its ability to mimic natural infection to trigger a more potent immune response and rapid production. The phase I clinical trial has recently commenced at the Kaiser Permanente Washington Health Research Institute in Seattle, USA. However, the availability of this vaccine for global immunization is likely to take more time as it faces numerous challenges associated with clinical development, since no previous mRNA vaccine has been approved. The second vaccine, the Ad5-nCov, a replication-defective adenovirus vector expressing the SARS-CoV-2 spike protein, has been developed jointly by Cansino Biologics Inc. (Tianjin, China) and the Beijing Institute of Biotechnology (Beijing, China). The Sartorius’ Biostat STR single-use bioreactor system was used for the upstream preparation of the recombinant vaccine. The phase I clinical trial of this vaccine candidate recently commenced at the Tongji Hospital in Wuhan, China [123].

The phase I clinical trials for two other vaccine candidates (BNT-162 and INO-4800) are due in April 2020 in China and the USA, respectively. The BNT-162, an mRNA vaccine candidate, expressing codon-optimized undisclosed SARS-CoV-2 protein(s) encapsulated in lipid nanoparticles, has been jointly developed by Biontech AG (Mainz, Germany), Shanghai Fosun Pharmaceutical Co. Ltd. (Shanghai, China) and Pfizer Inc. (New York, NY, USA) [13] (Hodgson, 2020). Another candidate, the INO-4800, a DNA vaccine supported by CELLECTRA^®^ 3PSP, a hand-held smart device for intradermal delivery, developed by Inovio Pharmaceuticals Inc. (Plymouth Meeting, PA, USA) and Beijing Advaccine Biotechnology Co. Ltd. (Beijing, China) [13], is undergoing phase I clinical trials. The globally coordinated efforts to combat SARS-CoV-2 have led to the development of potential vaccine candidates in the shortest possible time; however, these candidates need to be subjected to rigorous clinical trials to prove their efficacy and safety before being considered for global immunization. Therefore, in the current situation, therapeutic drugs are the most effective alternative for the containment of COVID-19 and restoration of public health. Recently, two more vaccine candidates also entered phase I clinical trials, the details of which are summarized in Table 5.

A concept of protection through changing the viral genetic code was given by researchers from China in 2016 while working on influenza A virus. They generated premature termination codons (PTCs), and these PTC-containing viruses showed promising results in mice, guinea pigs, and ferrets against influenza challenge. However, such studies have not been replicated further, though in the hour of need during the COVID-19 crisis, must be evaluated for the beneficial and effective control of infections.

### 13.2. Development of Antiviral Therapeutics

The rapid spread of SARS-CoV-2 demands the immediate development of effective and safe therapeutic strategies. In the absence of licensed vaccines and approved antiviral drugs, the most promising approach adopted by researchers is the repurposing of drugs [12,124]. However, no specific anti-SARS-CoV-2 treatment has been recommended by the US FDA, yet owing to the lack of supportive data.

Favilavir (Favipiravir), the first antiviral drug tested against SARS-CoV-2, approved by the National Medical Products Administration of China in February 2020, is commonly used to treat influenza infection in China and Japan and has shown promising results in shortening the course of SARS-CoV-2 infection [12,124,125]. This drug acts by inhibiting the RNA-dependent RNA polymerase activity (RdRp) of SARS-CoV-2. Despite its potential effectiveness, the drug is yet to be approved by the U.S. FDA. Remdisivir has also been reported to treat COVID-19 patients to full recovery [126,127]. In fact, remdisivir is also a repurposed drug that was initially developed for the treatment of Ebola virus infection. Remdisivir is reported to prevent viral replication by the premature termination of RNA transcription. Currently, remdisivir is under clinical trials for the treatment of COVID-19 patients. Furthermore, a combination of lopinavir and ritonavir has been reported to significantly improve the conditions of COVID-19 patients in South Korea [126]. Similar to remdisivir, a combination of lopinavir and ritonavir was developed to treat HIV-1 infections in adults. However, the combination of lopinavir and ritonavir failed to serve its initial purpose when used for the treatment of COVID-19 patients in recent clinical trials in China [128]. Hydroxychloroquine, an anti-malarial drug, in combination with azithromycin, has shown promising results in an open-label non-randomized clinical trial in reducing the viral load in COVID-19 patients [129]. However, its associated side-effects could be more severe in COVID-19 patients with pre-existing chronic medical conditions.

Apart from these drugs, convalescent plasma therapy (plasma from recovered COVID-19 patients that contains antibodies against SARS-CoV-2) could also be used as an alternative treatment strategy to improve the survival rate of COVID-19 patients [130]. Recently, the FDA allowed the use of plasma therapy for critically ill patients under an emergency investigational new drug protocol (FDA, 2020). The treatment strategies must focus on curbing hyper inflammation, and the existing readily available therapies may prove beneficial in patients with severe SARS-CoV-2 infection. A humanized monoclonal antibody, viz. tocilizumab, competitively binds to the IL-6 receptors and subsequently prevents the binding of IL-6 to its receptor [131]. Notably, the blocking of the IL-6 receptor by tocilizumab may prove to be crucial and reduce the number of deaths associated with severe stage COVID-19. In this context, a clinical trial (ChiCTR2000029765) using tocilizumab reported its significant effectiveness in controlling fever and improving the respiratory function in individuals with severe COVID-19 [98]. Apart from the aforementioned drugs, there are several other drugs that are either under clinical trials or are being considered for a clinical trial for the treatment of COVID-19. Of note, the information provided in this study is neither exhaustive nor should be used for self-medication.

### 13.3. Challenges of COVID-19 Vaccines and Therapeutics

With the global cohesive approach, dozens of vaccine candidates have reached a status needing human trials. The human trials can only provide an idea on the status of vaccine effectiveness. The search for volunteers is a challenge, though in few places a number of people have registered for the clinical trials. The time estimated for the release of any vaccine looks lengthier, with a waiting period of nearly 12 months, involving quality checks, regulatory, and scale-up issues, with the required number of doses covering those who are suffering globally. A few of the candidate vaccines cleared the animal trials with safe outcome and need an assessment of immune responses and disease enhancement. Lately, other concerns posing a challenge are higher mutability seen in circulating strains of SARS-CoV-2 worldwide and the antibody-dependent enhancement (ADE) of disease. This ADE mechanism enables the entry of the virus into the host cells and is well described in viral infections. “Will the changing virus remain susceptible to the upcoming vaccine?” and “Could it be helping in ADE?” are the questions yet to be resolved.

## 14. Mitigation Strategies

In the absence of approved antiviral agents and vaccines, the most effective strategies are based on the disruption of the transmission cycle of the virus (human-to-human) via droplets or close contact. To achieve this, the most critical step would involve maintaining strict hand hygiene and adherence to basic cough etiquettes, across all strata from children to the elderly. A study reported the employment of prevention and control strategies at three levels: case-related population, general population, and national level [132]. Additionally, the National Health Commission of China issued protocols for the mitigation of COVID-19 in order to contain the further spread of the virus by a “big isolation and big disinfection” policy during the Chinese spring festival [133]. A national-level mitigation strategy has also been adopted for the elderly population and in rural areas [134,135]. Awareness regarding the connotations of hand hygiene amongst the general public, instead of stressing the usage of masks, is the need of the hour in view of the multiple modes of transmission. The use of sanitizers or soaps is one of the essential components of the prevention and control of COVID-19. Sanitizers containing 60–70% ethanol or 70% isopropanol are effective against SARS-CoV-2. Similarly, household detergents or soaps are also useful in sanitization. While sanitizers are sufficient for relatively clean hands, soiled hands need to be washed using soap and water for 20 s. Additionally, the WHO issued guidelines on the use of face masks during homecare visits and in the community, along with health care settings in the context of COVID-19 [136]. Moreover, the use of particulate respirators, such as certified N95 or FFP2, is recommended for health care workers who perform aerosol-generating procedures.

In contrast, medical masks are recommended while providing clinical care to suspected or confirmed COVID-19 cases [136]. The WHO has approved the use of N95 masks for patients at all times for source control and for healthcare providers only in certain situations, such as when they are in the close vicinity of a patient (within three feet) or are performing aerosol-generating procedures on symptomatic patients. These masks are not recommended for use by the general public. In situations of close contact (within three to six feet) with a suspected or confirmed case, a facemask ought to be used instead of an N95 or powered air-purifying respirator. If the patient is not using a facemask, the health care provider should wear an N95 mask, even when at a distance of three to six feet from the patient. In low-resource outbreak settings, the reuse of N95 masks after ethylene oxide sterilization may be considered. General public awareness should be increased by the display of posters with clear “DO’s” and “DON’Ts,” illustrating symptoms, routes of transmission, and preventive measures, with emphasis on personal hygiene to curtail transmission. Physical and social distancing campaigns should be promoted to lessen physical contact and to contain the spread. Fitbit devices and other smartphone fitness devices can be used during such outbreaks to monitor the symptoms [137,138]. Smartphones and internet services can also be utilized to disseminate information regarding the presentation and prevention of the infection.

Disposable protective clothing, eye gear, gloves, masks, and shoe covers should be used while handling animals and animal products, and the same should be restricted to the workplace to prevent the spread to family members. The consumption of undercooked and raw meat should strictly be avoided. The proper heating of food to a temperature of 60 °C for thirty minutes, mimicking thermal shock conditions, a common practice in India, is sufficient to kill the virus. Breath analyzers and other public sharing equipment should use a disposable mouthpiece to prevent the human-to-human transfer of the virus. The proper cleaning of all surfaces should be practiced in hospitals, airports, at workplaces, and in domestic settings to allow for the appropriate action of detergents since SARS-CoV is an enveloped virus. The virus has been found to persist on inanimate surfaces from two hours up to nine days at room temperature, depending upon the type of surface. At temperatures of 4 °C, certain strains remain viable for up to 28 days. Surface disinfection with 0.1% hypochlorite or 62-71% ethanol was found to be effective in reducing the infectivity of a surface within an exposure time of one minute [139].

Unfortunately, this is the third event of the spillover of a zoonotic respiratory virus to humans in this decade of the 21st century, highlighting the necessity of prerequisite multidisciplinary coordination for curbing the emergence of novel viruses and controlling such apocalyptic events [140,141,142]. The “One health approach” is one such initiative that incorporates preventive strategies for the emergence of zoonosis in all three arms: humans, animals, and the environment [143,144]. WHO initiatives, such as the Battle against Respiratory Viruses (BRaVe), that support innovative multidisciplinary research in the field of emerging and re-emerging respiratory viruses are encouraging [145]. Consortiums and open-access platforms, such as ProMed, International Severe Acute Respiratory and Emerging Infection Consortium, and BioDiaspora, act as benefactors by partaking in, stipulating, and disseminating comprehensive knowledge on the prevention and management of such events worldwide.

Travelers from COVID-19 affected countries need to be quarantined appropriately, as they have been the root cause of spreading SARS-CoV-2 from China to subsequently more than 200 countries. Measures, such as public awareness programs about COVID-19 associated health risks and how to avoid contact transmission, issuing updated advisories from time to time, visa cancellations to avoid travelers coming in from affected countries, understanding the high socio-economic implications of SARS-CoV-2 with a follow up of suitable ameliorating measures, closing schools, offices, malls, and other public places, staying at home in affected areas during lockdown conditions in countries where this virus has been severely affected, strengthening medical facilities, adopting high biosafety measures for doctors, nurses, and health-care workers, along with following high standards of hygiene and health practices while diagnosing and treating COVID-19 patients, are a few of the critical prevention and control measures to check the high spread of this pandemic virus.

Because of the current pandemic situation affecting more than 200 countries, as well as the global threats imposed by SARS-CoV-2/COVID-19, as indicated by the increasing number of cases and deaths, researchers are working to develop effective vaccines and therapeutics as soon as possible. Appropriate surveillance, prevention, and control measures are being implemented worldwide, while taking lessons from earlier virus outbreaks, such as SARS-CoV, MERS-CoV, Ebola, Zika, Nipah, and others, as well as from the advances in science and technology in the modern era, to halt the further spread of this virus and to restrain COVID-19 [118,146,147,148,149,150,151,152,153,154].

## 15. Conclusions

The emergence of SARS-CoV-2 highlights gaps in the current knowledge about human–animal barriers and zoonotic diseases, as well as the necessity for a prerequisite for extensive comprehensive research at the human–animal–environment interface of the One Health concept [155], to determine the origins and crossroads of interspecies transfer impediment by the identification of the ecological and evolutionary carriers of the infection. Several questions on SARS-CoV-2 remain unanswered, including the factors behind the virus crossing the species barrier, the definitive origin of the virus, the exact roles of distinct ORFs, the critical point mutation differences in virus transmission and pathogenesis, the reason for the death of specific infected individuals while others remain asymptomatic, and the reappearance of infection in specific cured individuals, among others. With the availability of more complete genome sequences from different parts of the world, it is becoming apparent that several virus mutants are circulating and need close monitoring. There is a need to identify the role of the ADE mechanism in SARS-CoV-2, as well to plan for better immunomodulatory therapies for the effective treatment and control of COVID-19 infections.

## Figures and Tables

**Table 1:** Comparison of the typical features of human coronaviruses isolated between 2002–2020 (SARS-CoV-2, MERS-CoV, and SARS-CoV).

Features	SARS	MERS	COVID-19
Causative agent	SARS-CoV	MERS-CoV	SARS-CoV-2
Incubation period	2–10 days	2–10 days	2–14 days
The median age of infected human cases	65 years	50 years	59 years
Source of origin	Bats, civet cats	Bats, camel	Seafood, bats, pangolin (proposed)
Transmission	Animal–human Human–human Zoonotic disease	Animal–human Human–human Zoonotic disease	Animal–human Human–human Human–animal Zoonotic disease
Speed of spread	Moderate	Low	High
Seasonal occurrence	Winter (Dec–Jan)	Summer (May–July)	Winter (Dec–Jan)
Place of Origin	Guangdong, China	Jeddah, Saudi Arabia	Wuhan, China
First incidence	16 November 2002	13 June 2012	7 December 2019
The last case reported and present status	5 July 2003	18 February 2020 (Ongoing)	Ongoing
Total number of cases	8422	2496	1,434,426 as of 8 April 2020
Overall fatality	916 (10.87%)	868 (34.77%) (as of now)	82,220 (5.73%) as of 8 April 2020
No. Countries affected	31	27	212 (till now)
Intermediate host	*Paguma larvata*	*Camelus dromedaries*	Pangolin, Mink (possible)
Definitive host	*Rhinolophus sinicus*	*Pipistrellus hesperidus*	*Rhinolophus affinis* (possible)
Taxonomy	*Betacoronavirus* (*Serbecovirus*)	*Betacoronavirus* (*Merbecovirus*)	*Betacoronavirus* (*Serbecovirus*)
Genome length (bases)	29751	30119	29903
Major Regional distribution	Guangdong province of southern China, and later to western pacific countries	Saudi Arabia, followed by UAE and Republic of Korea	Hubei, especially, Wuhan in China, followed by worldwide
Treatment/Vaccine	Glucocorticoid and Interferon	No effective approved treatment or vaccine	Lopinavir/Ritonavir (in testing)
Receptor	Angiotensin-converting enzyme-2 (ACE-2)	Dipeptidyl peptidase 4 (DPP4)	ACE-2

**Table 2:** Receptors recognized by prototype CoVs from different genera.

Genus	CoVs	Receptors
*Alphacoronavirus*	HCoV-NL63	ACE-2
	TGEV	APN
	PRCV	APN
*Betacoronavirus*	MHV	CEACAM1
	BCoV	N-acetyl-9-O-acetylneuraminic acid
	MERS	DPP4 (CD26)
	SARS	ACE-2
*Gammacoronavirus*	IBV	α2,3-linked sialic acid (attachment factor)
*Deltacoronavirus*	PDCoV	APN

**Table 3:** A summary of (a) percentage nucleotide and (b) protein sequence identities of SARS-CoV-2 (NC_045512, Wuhan-Hu-1) with other members of *Betacoronavirus* (subgenus—*Sarbecovirus*).

CoVs	Strain Name/ Accession Number	% Nucleotide Sequence Identity	% Protein Sequence Identity
**(a)Whole Genome-Based Comparison (29.9kb)**			
South Korea	SNU01/MT039890	99	99.9
Australia	VIC01/MT007544	99	99.9
Nepal	61-TW/MT072688	99–100	100
Taiwan	NTU02/MT066176	99–100	100
USA	USA-CA9/MT118835	99–100	100
Japan	WK-501/LC522974	99–100	100
**(b) Spike Protein-Based Comparison (~3800 bases)**			
Pangolin CoV	MP789/MT084071	89.7	89.78
Bat_SL_CoVs	bat-SL-CoVZC45/MG772933 bat-SL-CoVZXC21/MG772934	77.5–78.3	81.2–81.8
Bat_CoV	RaTG13/MN996532	93.1	97.69
Bat_SARS_CoVs	WIV16/KT444582 BtRs-BetaCoV/YN2018C/MK211377	69.8–73.9	72.3–78.1
SARS_CoVs	NS-1/AY508724 GD01/AY278489	73.9	77
MERS_CoVs	HCoV-EMC/JX869059 England 1/NC_038294	21.8–21.9	26.8
CRCoVs	CRCVK39/EU983107 Respiratory/AY150272	23.1–23.2	28.8–29.1
BCoVs	CAMAGUEY/HE616741 HT317/MK046011	18.2–22.7	16.9–28.7
EqCoV	Obihiro12-1/LC061273	18.3	29.2
HCoV	OC43/S62886	18.1–18.7	26.8–28.0
Bat_CoV	Zhejiang2013/KF636752	33.6	39.0
Bat_CoVs	KHU9-1/EF065513 GCCDC1 356/KU762338	24.3–25.1	29.1

**Table 4:** A summary of selected molecular diagnostic tests that received emergency use authorization (EUA) from regulatory bodies.

Company	Assay	Targets	Regulatory Status
Centers for Disease Control and Prevention (CDC)	CDC 2019-nCoV Real-Time RT-PCR Diagnostic Panel (CDC)	N1, N2 and RNase P (control)	USA FDA-EUA
Roche Molecular Systems, Inc. (RMS)	cobas SARS-CoV-2	ORF1ab, E gene, RNase P (control)	USA FDA-EUA
Thermo Fisher Scientific, Inc.	TaqPath COVID-19 Combo Kit	ORF1ab, N gene, S gene, MS2 (control)	USA FDA-EUA
Primerdesign Ltd.	Primerdesign Ltd. COVID-19 genesig Real-Time PCR assay	RdRp gene	USA FDA-EUA
Abbott Molecular	Abbott RealTime SARS-CoV-2 assay	RdRp and N genes	USA FDA-EUA
Cepheid	Xpert Xpress SARS-CoV-2 test	N2 and E genes	USA FDA-EUA
DAAN Gene Co., Ltd. of Sun Yat-sen University	Detection Kit for 2019 Novel Coronavirus (2019-nCoV) RNA (PCR-Fluorescence Probing)	ORF1ab and N gene	China EUA
Seegene, Inc.	Allplex 2019-nCoV assay	E, RdRp and N genes	Korea EUA

**Table 5:** A summary of vaccine and drug candidates being used or under consideration (phase I clinical trials) for the prevention/treatment of COVID-19.

Vaccine/Drug Candidates	Developer	Status	Remarks
**Vaccines candidates**			
mRNA-1273	Moderna/NIAID	Phase 1 (NCT04283461)	Clinical trials started at Kaiser Permanente Washington Health Research Institute in Seattle, USA
Ad5-nCov	CanSino Biological Inc. and Beijing Institute of Biotechnology	Phase 1 (ChiCTR2000030906)	Clinical trials recently started at Tongji Hospital in Wuhan, China
INO-4800	Inovio Pharmaceuticals	Phase I (NCT04336410)	DNA plasmid encoding S protein delivered by electroporation
LV-SMENP-DC	Shenzhen Geno-Immune Medical Institute	Phase I (NCT04276896)	Dendritic cells modified with lentiviral vector and expressing synthetic minigene based on domains of selected viral proteins
Pathogen-specific aAPC	Shenzhen Geno-Immune Medical Institute	Phase I (NCT04299724)	Artificial antigen-presenting cells (aAPCs) modified with lentiviral vector and expressing synthetic minigene based on domains of selected viral proteins
**Drug candidates**			
Favilavir	Hisun Pharmaceutical, China	Approved by NMPA, China	Inhibition of RNA-dependent RNA polymerase of SARS-CoV-2
Hydroxychloroquine and azithromycin	USA	Clinical trials are under progress	Both have shown in vitro activity against SARS-CoV-2
Remdesivir	Gilead Sciences, USA	Clinical trials are under progress	Developed for treatment of Ebola virus infection
Lopinavir + Ritonavir	AbbVie, USA	Further research is under process	Developed to treat HIV-1 infections

## Supplementary Materials

The following are available online at https://www.mdpi.com/2076-0817/9/7/519/s1, Table S1: The functions of non-structural proteins in a typical coronavirus and SARS-CoV-2 (predicted by bioinformatics tools).
