# Supplementary material for: Coronavirus Disease Pandemic (COVID-19): Challenges and a Global Perspective
Source: Pathogens. 2020 Jun 28;9(7):519. doi: 10.3390/pathogens9070519 (PMC7400054; doi:10.3390/pathogens9070519)
Supplement: Supplementary file 1 [file pathogens-09-00519-s001.pdf]

Supplementary Table 1: The functions of non-structural proteins in a typical coronavirus and SARS-CoV-2 (predicted by bioinformatics tools).

Non-Structural proteins	Functions of NSP in a Typical CoV	Functions of NSP in SARS-CoV-2
nsp1	Cellular mRNA degradation, inhibiting IFN signaling	Suppress antiviral host response
nsp2	Unknown	Unknown
nsp3	PLP, polypeptides cleaving, blocking host innate immune response, promoting cytokine expression	Putative PL-pro domain
nsp4	DMV (double-membrane vesicle) formation	Complex with nsp3 and 6: DMV formation
nsp5	3CLpro, Mpro, polypeptides cleaving, inhibiting IFN signaling	3CL-pro domain
nsp6	Restricting autophagosome expansion, DMV formation	Complex with nsp3 and 4: DMV formation
nsp7	Cofactor with nsp8 and nsp12	Complex with nsp8: primase
nsp8	Cofactor with nsp7 and nsp12, primase	Complex with nsp7: primase
nsp9	Dimerization and RNA binding	RNA/DNA binding activity
nsp10	Scaffold protein for nsp14 and nsp16	Complex with nsp14: replication fidelity
nsp11	Unknown	Short peptide at the end of orf1a (2019-nCoV HKU-SZ-005b)
nsp12	Primer dependent RdRp	RNA-dependent RNA polymerase
nsp13	RNA helicase, 5′triphosphatase	Helicase
nsp14	Exoribonuclease, N7-MTase	ExoN: 3′–5′ exonuclease
nsp15	Endoribonuclease, evasion of dsRNA sensors	XendoU: poly(U)-specific endoribonuclease
nsp16	2′-O-MTase; avoiding MDA5 recognition, negatively regulating innate immunity	2′-O-MT: 2′-O-ribose methyltransferase
